# PPRC1, but not PGC-1α, levels directly correlate with expression of mitochondrial proteins in human dermal fibroblasts

**DOI:** 10.1590/1678-4685-GMB-2019-0083

**Published:** 2020-07-03

**Authors:** Mateus Prates Mori, Nadja Cristhina de Souza-Pinto

**Affiliations:** 1 Universidade de São Paulo, Departamento de Bioquímica, Instituto de Química, São Paulo, SP, Brazil

**Keywords:** XPC, fibroblast, PGC-1α, PPRC1, mitochondria

## Abstract

The XPC protein, which is mutated in xeroderma pigmentosum (XP) complementation group C (XP-C), is a lesion recognition factor in NER, but it has also been shown to interact with and stimulate DNA glycosylases, to act as transcriptional co-activator and on energy metabolism adaptation. We have previously demonstrated that XP-C cells show increased mitochondrial H_2_O_2_ production with a shift between respiratory complexes I and II, leading to sensitivity to mitochondrial stress. Here we report a marked decrease in expression of the transcriptional co-activator PGC-1α, a master regulator of mitochondrial biogenesis, in XP-C cells. A transcriptional role for XPC in PGC-1α expression was discarded, as XPC knockdown did not downregulate PGC-1α expression and XPC-corrected cells still showed lower PGC-1α expression. DNA methylation alone did not explain PGC-1α silencing. In four different XP-C cell lines tested, reduction of PGC-1α expression was detected in three, all of them carrying the c.1643_1644delTG mutation (ΔTG) in XPC. Indeed, all cell lines carrying XPC ΔTG mutation, whether homozygous or heterozygous, presented decreased PGC-1α expression. However, this alteration in gene expression was not exclusive to XPC ΔTG cell lines, for other non-related cell lines also showed altered PGC-1α expression. Moreover, PGC1-α expression did not correlate with expression levels of TFAM and SDHA, known PGC-1α target-genes. In turn, PPRC1, another member of the PGC family of transcription co-activators controlling mitochondrial biogenesis, displayed a good correlation between its expression in 10 cell lines and TFAM and SDHA. Nonetheless, PGC-1α knockdown led to a slight decrease of its target-gene protein level, TFAM, and subsequently of a mtDNA-encoded gene, MT-CO2. These results indicate that PGC-1α and PPRC1 cooperate as regulators of mitochondrial biogenesis and maintenance in fibroblasts.

## Introduction

DNA repair disorders represent an important tool to understand the role of mutations in the development of diseases. Xeroderma pigmentosum (XP) patients carry mutations in genes encoding proteins of the nucleotide excision repair (NER) pathway. In humans, NER is the only DNA repair pathway with the ability to remove UV-induced DNA damage, more specifically cyclobutane pyrimidine dimers (CPDs) and 6,4-pyrimidine-pyrimidone photoproducts (6,4-PP), and consequently XP patients present markedly increased susceptibility to early onset of skin neoplasia on the exposed area (DiGiovanna and Kraemer, 2012). The disease is classified into eight complementation groups according to the mutated gene: XP-A to XP-G, with mutations in protein-coding genes of the NER pathway [*XPA, ERCC3* (XPB), *XPC, ERCC2* (XPD), *DDB2* (XPE – UV-DDB complex), *ERCC4* (XPF) e *ERCC5* (XPG)] and XP-V (XP variant), with mutations in the *POLH* gene coding the translesions synthesis DNA polymerase Pol η (Cleaver *et al.*, 2009).

XP complementation group C (XP-C) patients exhibit only the classical XP phenotype without distinguishable neurological symptoms (Cleaver *et al.*, 2009), although there are three or four exceptions in the literature (Rivera-Begeman *et al.*, 2007; Soufir *et al.*, 2010; Uribe-Bojanini *et al.*, 2017). The XPC protein is the major subunit of the heterotrimeric complex XPC-RAD23B-centrin 2, which is involved in NER in lesion recognition (Sugasawa *et al.*, 1998; Volker *et al.*, 2001). The XPC complex senses nucleobase lesions with disrupted canonical hydrogen bonding through delayed dissociation of DNA-XPC protein complex compared to the dissociation of DNA-protein complex in undamaged DNA (Min and Pavletich, 2007; Chen *et al.*, 2015).

In addition to its well-established role in NER, new roles for the XPC protein were proposed in the last decade. D'Errico *et al.* (2020). identified a functional interaction between XPC and oxoguanine DNA glycosylase (OGG1) – a DNA glycosylase of the base excision repair (BER) pathway. Experimental evidences indicated that XP-C cells are more sensitive to oxidatively-induced DNA damage that correlates not only with the delayed repair of 5’,8-cyclopurine – a NER substrate – but also of 8-oxo-7,8-dihydroxyguanine (8-oxoGua) – an OGG1 substrate of BER. Shimizu *et al.* (2003 and 2010) also showed that XPC interacts with two other DNA glycosylases of BER *in vitro*: TDG and SMUG1. The former was found to participate in the active demethylation of the oxidized form of the epigenetic DNA modification 5-methylcytosine (Cortázar *et al.*, 2011). This XPC-TDG interaction was further confirmed *in vivo* and is involved in epigenetic rewiring during induced-pluripotent stem cell reprogramming and it was also found in promoters of non-housekeeping genes after transcriptional activation in the absence of DNA damage (Le May *et al.*, 2010; Fong *et al.*, 2011; Ho *et al.*, 2017). In another study, Rezvani *et al.* (2011) reported a metabolic adaptation in keratinocytes constitutively silenced with shRNA for XPC. In that article the authors showed that XPC ablation induces a metabolic shift via activation of the DNA-PK/AKT1/NOX1 axis, with increased reliance on glycolysis over oxidative phosphorylation for ATP generation.

**Figure 1:**
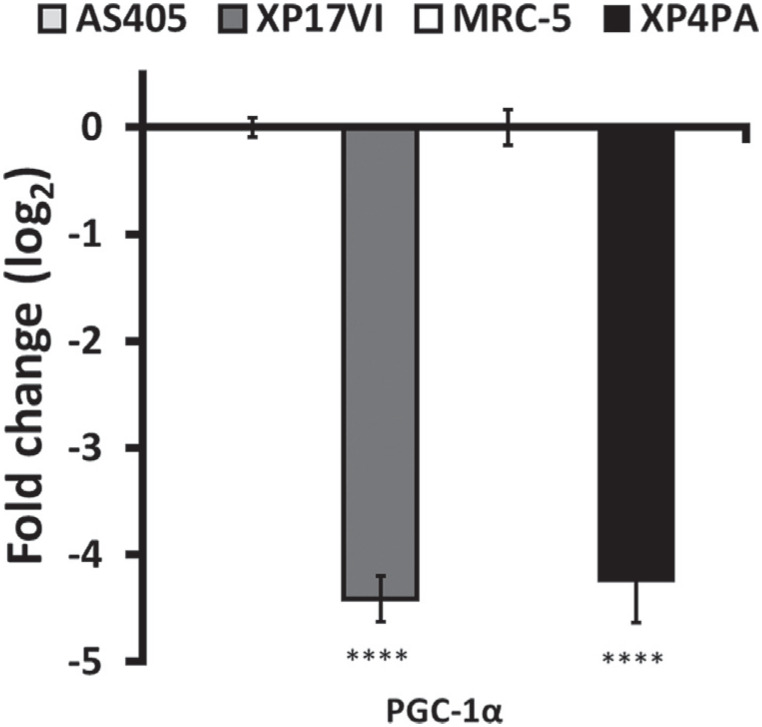
PGC-1α is differentially expressed in XP-C cells. PGC-1α expression levels were measured in the following cell lines: matched human primary fibroblasts, AS405 (WT) vs. XP17VI (XPC^Δ^TG/ΔTG), and SV-40 immortalized human fibroblasts, MRC-5 (WT) vs. XP4PA(XPC^ΔTG/ΔTG^). Results are shown as mean±SD, n=4. *p<0.05, **p<0.01, ***p<0.001 and ****p<0.0001.

We have recently reported that XP-C cells display a shift between respiratory complexes I and II utilization, accompanied with increased mitochondrial H_2_O_2_ production and decreased GPx activity (Mori *et al.*, 2017). Moreover, this shift between complex I and II resulted in an increased cellular sensitivity to mitochondrially induced redox unbalance that was fully reverted in an isogenic corrected XP-C cell line. In a screening panel of mitochondrial biogenesis and other mitochondrial-related genes we found PGC-1α to be surprisingly downregulated (Figure 1), which led us to postulate that XPC protein or mRNA levels modulated PGC-1α expression.

In the present study, we show that XP-C cell lines carrying a dinucleotide deletion (ΔTG, n.1747_1748delTG) present decreased mRNA levels of PGC-1α without any impact on mitochondrial biogenesis genes. We further demonstrate that, in fibroblasts, mRNA levels of the PGC-1-related coactivator PPRC1 correlate better with expression of mitochondrial genes. But although PGC-1α mRNA levels did not correlate with mRNA expression of two target genes, PGC-1α knockdown has a minor but significant impact on their protein levels. Together these results demonstrated that PGC-1α and PPRC1 work in concert to regulate mitochondria homeostasis in human fibroblasts.

## Material and Methods

### Cell lines and culture

The human SV40-transformed fibroblast cell lines MRC-5 (XPC^+/+^), XP4PA (XP-C ^ΔTG/ΔTG^) carrying the dinucleotide deletion n.1747_1748delTG in exon 9 (formerly c.1643-1644delTG) and corrected XP4PA (XP4PA^corr^ – XPC^+/+)^ (Dupuy *et al.*, 2013) were kindly provided by Dr. Carlos F. M. Menck, ICB, University of São Paulo (USP). The human primary fibroblasts cell lines AS405 (wild type); XP16HM16VI (father of XP17VI) and XP16HFVI (mother of XP17VI) both heterozygous for XPC-ΔTG (XPC^ΔTG/+^); and XP17VI, XP03SP, AS480 (XP-C^ΔTG/ΔTG^), XP02SP and AS860 (XP-C^mut/mut^) were also provided by Dr. Carlos F. M. Menck. XP02SP carries a homozygous c.1969G>T transversion on exon 9 leading to a premature stop codon p.Glu657X (Leite *et al.*, 2009). AS860 carries a homozygous c.658C>T transition on exon 6 leading to a premature stop codon p.Arg220X (Soufir *et al.*, 2010). The human primary fibroblasts cell lines FDH107, FDH111 and FDH113 (all XPC^+/+^) were kindly provided Dr. Silvya Stuchi Maria-Engler, FCF, USP. All primary cell lines were maintained in DMEM/high glucose supplemented with 20% FBS, penicillin 100 IU/mL and streptomycin 100 μg/mL, at 37 °C in a humidified atmosphere with 5% CO_2_ (standard conditions). All SV40-immortalized cell lines were maintained in the same conditions described above, but with 10% FBS. Cultures were routinely sub-cultured, by trypsinization, when reached up to 80-90% confluence.

### Citrate synthase activity

Citrate synthase activity was assessed as described by Hansford and Castro (1982). Reactions were performed in a 96-well plate format containing 200 μL reaction buffer and substrates [Tris-HCl 0.1 M, pH 8.1, acetyl-CoA 5 mM, DTNB 100 μM, oxaloacetate 250 μM Triton X-100 0.1% (w/v)]. Reaction started after adding 2 μL of whole cell extracts adjusted to 1.5 mg/mL protein concentration (3 μg/well of protein) and absorption kinetics was measure at 412 nm in a SpectraMax 190 reader (Molecular Devices©) with one reading every 19 s (15 s for acquisition, 3 s for shaking and 1 s for waiting) for 5 min at 30 °C. Enzymatic activity was estimated as formation of DNTB-CoA from CoA-SH released after acetyl-CoA and oxaloacetate condensation. Values were calculated from TNB molar extinction coefficient (ε=13.6×10^−6^ M^−1^×cm^−1^ ou 13.6 μM^−1^×cm^−1^) and adjusted to represent the amount of citrate formation in mol per min per mg of protein (nmol/min/mg).

### Plasmid and siRNA transfection

Approximately 2.5×10^5^ MRC-5 cells were seeded per well in 6-well plates and incubated for 24 h in standard conditions. Mission® esiRNA targeting human XPC (EHU033441) and control esiRNA targeting EGFP (EHUEGFP) (Sigma-Aldrich®) were transfected using Lipofectamine® RNAiMAX (Life Technologies^TM^) according to the manufacturer's instruction. To investigate the role of PGC-1α in fibroblast we transfected four shRNA-conaining plasmids targeting PGC-1α and one scramble control (pGFP-V-RS, Origene©) in MRC-5 cells using Escort^TM^ III Transfection Reagent (Sigma-Aldrich®) according to the manufacturer's instruction. Transfection efficiency was assessed visualizing GPF-positive in Nikon Eclipse TE300 fluorescence microscope. After transfection, cells were kept under selective pressure in culture medium with puromycin 1.5 μg/mL.

### RNA isolation, cDNA synthesis and quantitative RT-PCR

RNA was isolated from immortalized cell lines cultivated in 6-well plates or from human primary fibroblasts cultivated in 60 cm^2^ dishes using RNeasy Micro Kit (Qiagen©) or GenElute^TM^ Mammalian Total RNA Miniprep Kit (Sigma-Aldrich®), according to the manufacturer's instruction. To check RNA integrity, 500 ng RNA was submitted to 1% (w/v) agarose gel electrophoresis in TAE supplemented with 1% bleach (v/v) to inhibit RNase activity (Aranda *et al.*, 2012). High Capacity cDNA Reverse Transcription® (Applied Biosystems®) followed by treatment with DNase-free RNase H (5U/reaction– Ambion®) for 30 min at 37 °C were used to prepare high quality cDNA from isolated RNA.

Total cDNA from cell lines was used to analyze the following genes: PGC-1α (*PPARGC1A*), PPRC1, SDHA, TFAM, XPC, and β-actin (*ACTB*) as the loading control. Three other housekeeping genes were compared to *ACTB* expression (*TUBB, TBP* and *HPRT*) none of which showed significant difference (not shown). For 5-aza-dC treated cells, we assessed KRT8 expression as a positive control (Liang *et al.*, 2002). Quantitative RT-PCR (RT-qPCR) reactions were performed using the Power SYBR Green PCR Master Mix^®^ (Applied Biosystems^®^), according to manufacturer's instructions, using 100 ng template and 5 pmol of each primer. Reactions and data collection were performed in a 7300 Real Time PCR System (Applied Biosystems®). Ct was manually set to 0.3 in all analysis and calculated according to ΔΔCt (Pfaffl, 2001) and adjusted as fold change (=log_2_^ΔΔCt^) accordingly. The primers sequences are presented in Table S1.

### Western blotting

Protein extracts were prepared from immortalized cell lines cultivated in 22 cm^2^ dishes or from human primary fibroblasts cultivated in 60 cm2. Cells were loosen using cell scrapper (TPP®) in ice cold PBS, centrifuged at 8,000 *g* for 1 min at 4 °C and supernatants removed. Cell pellets were incubated with RIPA lysis buffer [Tris-HCl 50 mM, pH 7.4, NaCl 150 mM, SDS 0.05% (w/v), sodium deoxycholate 0.5% (w/v), NP-40 0.5% (v/v)] supplemented with cOmplete^TM^ Mini Protease Inhibitor Cocktail (Roche®) in ice for 30 min followed by sonication (3 cycles of 15 s with intervals of 45 s with 20% amplitude). Cell lysates were centrifuged at 16,000 *g* for 10 min at 4 °C and supernatants transferred to a new microtube. Protein concentrations were estimated using Bradford Reagent and BGG as standard (both Bio-Rad®) and absorption reading at 595 nm in SpectraMax 190 reader (Molecular Devices©) in 96-well plate.

Protein samples were submitted to SDS-PAGE in 12% polyacrylamide gels and transferred to PVDF membranes. Membranes were stained in Ponceau S Staining solution [Ponceau S 0.1% (w/v), acetic acid 5% (v/v)] for 5 min, and de-stained washing twice in acetic acid 5% (v/v) for 5 min and twice in deionized water for 5 min. Membranes were block in BSA 5% (w/v) in TBS-T buffer [Tris-Cl 20 mM, pH 7.4, NaCl 150 mM, Tween® 20 0.05% (v/v), 0.02% sodium azide (w/v)] for 1 h at room temperature. Incubations with primary antibodies were performed in BSA 1% in TBS-T overnight at 4 °C, with the following antibodies: anti-MTCO2 [12C4F12] (1:500; mouse ab110258, Abcam®), anti-UQCRC2 [13G12AF12BB11] (1:500; mouse ab14745, Abcam®) and anti-TFAM (D5C8) (1:500; rabbit mAb #7495, Cell Signaling©). After washing thrice with TBS-T, membranes were incubated with anti-mouse IgG (1:15,000; goat IRDye® 800 S/N, 926-32210, LI-COR®) or anti-rabbit IgG (1:15,000; donkey IRDye® 680 S/N 926-68073, LI-COR®) in 1% milk in TBS-T for 1 h. Membranes were then washed thrice with TBS-T and visualized using Odyssey Infrared Imaging System (LI-COR®). Band intensity was calculated using ImageJ. Protein loading was normalized for total Ponceau staining.

### Statistical analysis

All statistical analysis was performed in GraphPad Prism 7. One-way ANOVA with Dunnet's post-test were performed for multiple comparisons. All ANOVA statistical analyses did not show any variation of standard deviation and, therefore, were homoscedastic (p>0.05). Student's *t*-test was performed for AICAR-treated samples (MRC-5 untreated vs. treated, and XP4PA-untreated vs. treated). Pearson's correlation coefficient (r) was calculated in GraphPad Prism with significance set to 95%. Multiple correlation coefficient (R) was calculated according to the following formula (Neter *et al.*, 1996):

$$R=\sqrt{\frac{r_{xz}^{2}+r_{yz}^{2}-2r_{xz}r_{yz}r_{xy}}{1-r_{xy}^{2}}}$$

where *r*_*xz*_, *r*_*yz*_, *r*_*xy*_ are Pearson's correlation coefficient. Here *x* (TFAM) and *y* (SDHA) are viewed as the independent variables (effect) and *z* (PPRC1 or PGC-1α) is the dependent variable (cause). The adjusted Multiple Coefficient of Determination (R^2^_adj_) was calculated according to the following formula (Neter *et al.*, 1996):

$$R_{adj}^{2}=1-\frac{\left(1-R^{2}\right)\times (n-1)}{n-k-1}$$

where *k* is the number of independent variables (2 – two) and *n* is the number of data elements in the sample for z (8 – eight).

## Results

### PGC-1α expression is sharply decreased in XP-C cell lines

In a previous study, our group demonstrated that XP-C cells shift electron transport chain (ETC) complexes usage, with increased complex II and decreased complex I-driven electron transport. This was completely reversed upon re-introduction of wild-type (WT) XPC *in locus* (Mori *et al.*, 2017), indicating a direct role for XPC in the metabolic adaptation. In that study, we detected only a slight upregulation in NRF1 and SIRT3 expression, which was consistent in both immortalized and primary XP-C fibroblasts (XP4PA and XP17VI) compared to their WT counterpart (MRC-5 and AS450). As NRF1 and SIRT 3 have more specific roles in controlling expression of mitochondrial proteins, we also measured mRNA levels of PGC-1α, a transcriptional co-activator known as a master regulator of mitochondrial biogenesis. While we observed small changes in most of the genes tested, often inconsistent between the transformed and primary pairs of cell lines, PGC-1α expression was markedly decreased (Figure 1). This result prompted us to investigate whether XPC was directly involved in PGC-1α regulation.

### XPC silencing, genetic correction of XP-C cells and DNA methylation do not affect PGC-1α expression in immortalized fibroblasts

To check whether PGC-1α expression level was dependent on XPC protein level we performed transient esiRNA knockdown in MRC-5 cells targeting XPC. Control EGFP-target esiRNA transfection did not affect XPC expression, as expected (Figure S1A). XPC-target esiRNA led to a 2.4-fold decrease in XPC gene expression (42.6%) without, however, affecting PGC-1α expression. Furthermore, genetic correction of XP4PA cell line in the XPC *locus* (XP4PA^corr^) did not restored PGC-1α expression as well (Figure S1B).

Barrès *et al.* (2010) demonstrated that DNMT3B-mediated non-CpG island methylation in the promoter region of PGC-1α altered its gene expression and, consequently, mitochondrial density in skeletal muscle cells. Thus, we treated MRC-5 and XP4PA cells with pan-DNA demethylating agent 5-aza-2’-deoxycytidine (5-aza-dC). As control, we measured expression levels of KRT8, which is overexpressed in fibroblasts after treatment with 5-aza-dC (Liang *et al.*, 2002). After an 8 day-treatment with 5-aza-dC 1 μM, both cell lines showed an increase in KRT8 expression when compared to negative control treatment (p<0.05). However, DNA demethylation alone did not affect PGC-1α expression in both cell lines (Figure S1C). These results indicate that XPC protein levels and DNA methylation in PGC-1α promoter region are not involved in PGC-1α silencing in XP-C cell lines.

### XP-C cell lines carrying XPC ΔTG mutation present decreased PGC-1α expression

**Figure 2:**
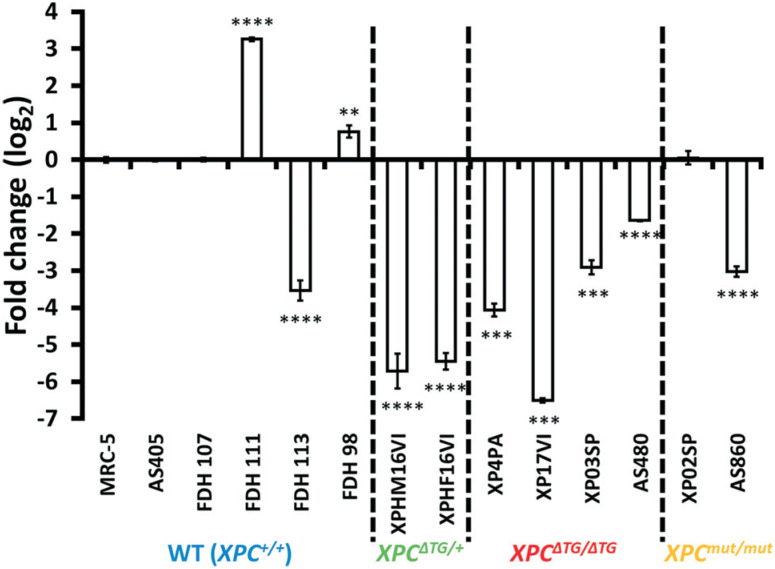
Analysis of PGC-1α gene expression in 14 cell lines. PGC-1α expression of human primary fibroblasts and SV-40 immortalized human fibroblasts were accessed by RT-qPCR. XP4PA was matched with MRC-5 (mean±SD, n=4). Fibroblasts FDH98 (female), FDH111, FDH113, XPHM16VI, XP17VI (all male) were matched with FDH107 (male) (mean±SD, n=4). Male fibroblasts XP02SP and XP03SP were matched with AS405 (mean±SD, n=3). Female fibroblasts XPHF16VI, AS480 and AS860 were matched with FDH98 (mean±SD, n=3). ***p<0.001.

Since both XP-C cell lines tested before, XP4PA and XP17VI, carry the same homozygous XPC ΔTG mutation, we tested whether a specific mutation in XPC could be driving PGC-1α silencing in the XP-C cell lines. Initially, samples were expanded to include two additional primary fibroblasts derived from XP-C patients: i) XP03SP (XP-C^ΔTG/ΔTG^), carrying the same homozygous ΔTG mutation, and ii) XP02SP (XP-C^mut/mut^) carrying a homozygous c.1969G>T transversion in exon 9 (Leite *et al.*, 2009). As shown in Figure 2, XP03SP carrying XPC ΔTG mutation also displayed reduced PGC-1α expression, although to a lesser extent. Interestingly, XP02SP carrying a different mutation showed unaltered PGC-1α expression compared to AS405.

To further explore the hypothesis that PGC-1α expression was modulated in a ΔTG XPC mutation-specific fashion, heterozygous (XPC^ΔTG/+^) cell lines (XP16HMVI and XP16HFVI, parents of individual XP17VI) were tested to check PGC-1α expression level. Surprisingly, PGC-1α expression levels were also diminished in both XPC^ΔTG/+^ parental cell lines (maternal XP16HFVI cell line expression was corrected and compared to FDH98 cell line, paired for sex).

### Decreased PGC-1α expression has no impact in mitochondrial content in XP4PA cells

PGC-1α expression is generally regarded as a predictor of mitochondrial content, as this transcriptional co-activator is believed to have a central role in the mitochondrial biogenesis program. We assessed citrate synthase activity as a surrogate for mitochondrial content and found that XP4PA cells showed similar mitochondrial content when compared to MRC-5 (Figure S2A), despite having a several-fold decrease in PGC-1α. Furthermore, the WT XPC corrected cell line (XP4PA^corr^), also have similar mitochondrial content, suggesting that PGC-1α expression does not correlates with mitochondrial content in fibroblasts.

### PGC-1α expression varies in human primary fibroblasts irrespectively of XPC and does not correlate with PGC-1 family of transcriptional coactivators target-genes

To further investigate the relationship between XPC and PGC-1 α expression, we added another three controls and two unrelated XP-C cell line, totalizing fourteen cell lines: i) six XP-C cell lines (four XPC^ΔTG/ΔTG^ and two XPC^mut/mut^); ii) two parental (XPC^ΔTG/+^) and; iii) six controls (one female and five male). After setting FDH107 as control reference for all male primary cell lines, FDH98 as control reference for all female primary cell lines, and MRC-5 as control reference for all immortalized cell lines, we observed a significant variation in PGC-1α expression levels regardless of XPC status (mutated or not) (Figure 2). Considering that the data are expressed in fold-change (y=log_2_), the variation between the PGC-1α top high expressing (FDH111) and top low expressing (XP17VI) was approximately 10, i.e., almost three orders of magnitude.

**Figure 3:**
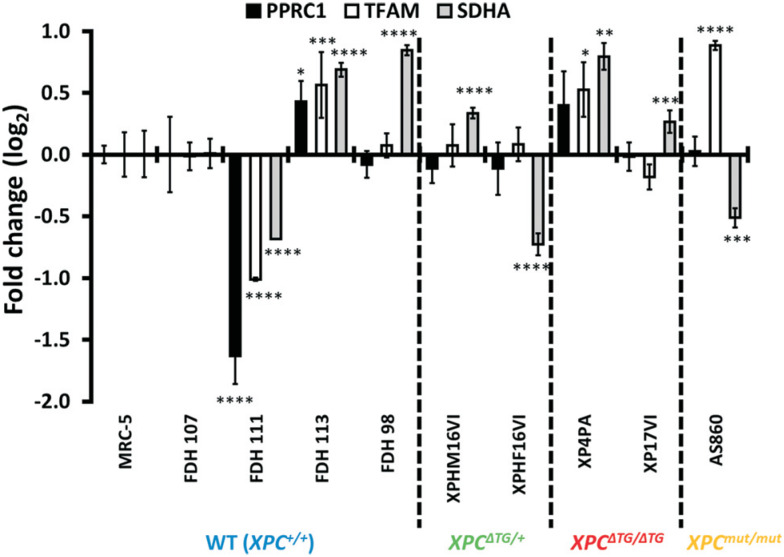
Analysis of PPRC1, TFAM and SDHA gene expression in 10 cell lines. PPRC1, TFAM and SDHA mRNA expression of human primary fibroblasts and SV-40 immortalized human fibroblasts were accessed by RT-qPCR. XP4PA was matched with MRC-5 (mean±SD, n=4). Fibroblasts FDH98 (female), FDH111, FDH113, XPHM16VI, XP17VI (all male) were matched with FDH107 (male) (mean±SD, n=4). Female fibroblasts AS860 and XPHF16VI were matched with FDH98 (mean±SD, n=3). *p<0.05, **p<0.01 and ***p<0.001.

To check if this variation (Figure 2) is biologically relevant, we investigated the expression levels of two genes under PGC-1α transcriptional control, TFAM and SDHA (Figure 3). We calculated Pearson's correlation between gene expression of PGC-1α vs. TFAM or SDHA for each cell line. Result scatter plots from eight cell lines (Figures S2B, C) yielded no significant correlation between PGC-1α expression *vs.* TFAM or *vs.* SDHA levels (p=0.2208 and p=0.7270, respectively), suggesting that in this cellular model another transcriptional coactivator may be involved in mitochondrial biogenesis.

### PPRC1 is more abundantly expressed in fibroblasts than PGC-1α and β and better correlates with PGC-1 family of transcriptional coactivator target-genes

The PGC-1 family of transcriptional coactivators comprises 3 members, PGC-1α, PGC-1β and PPRC1. We analyzed their expression levels by organ, tissue and cell type using the GTEx Portal at UCSC genome browser and Expression on PubMed Gene Database. Because expression data on PubMed did not include fibroblast, only GTEx Portal data were taken into consideration.

The top 3 PGC-1α expressing organs, tissues and cells were thyroid (transcripts per million, TPM=17.7), heart (atrial appendage) (TPM=13.86) and skeletal muscle (TPM=11.5) among 53 samples analyzed on GTEx Portal (Figure S3A). Transformed fibroblasts presented a median of 1.46 TPM, 8-12 times less than the top 3 expressing tissues. The top 3 PGC-1β expressing organs, tissues and cells were brain (cerebellum) (TPM=10.34), brain (cerebellar hemisphere) (TPM=9.94) and colon (transverse) (TPM=9.43) (Figure S3B). Transformed fibroblasts presented a median of 0.59 TPM, 16-18 times less than those. On the other hand, the top 3 PPRC1 expressing organs, tissues and cells were fallopian tube (TPM=35.49), ovary (TPM=35.45) and EBV-transformed lymphocytes (TPM=32.97) (Figure S3C). Surprisingly, transformed fibroblasts presented a median of 30.38 TPM, near to the top 3 expressing samples. Indeed, PPRC1 seemed to be more ubiquitously expressed among tissues since the difference between the highest and lowest expressing tissues was around 9X TPM *vs.* 36X for PGC-1β and 590X for PGC-1α.

**Figure 4:**
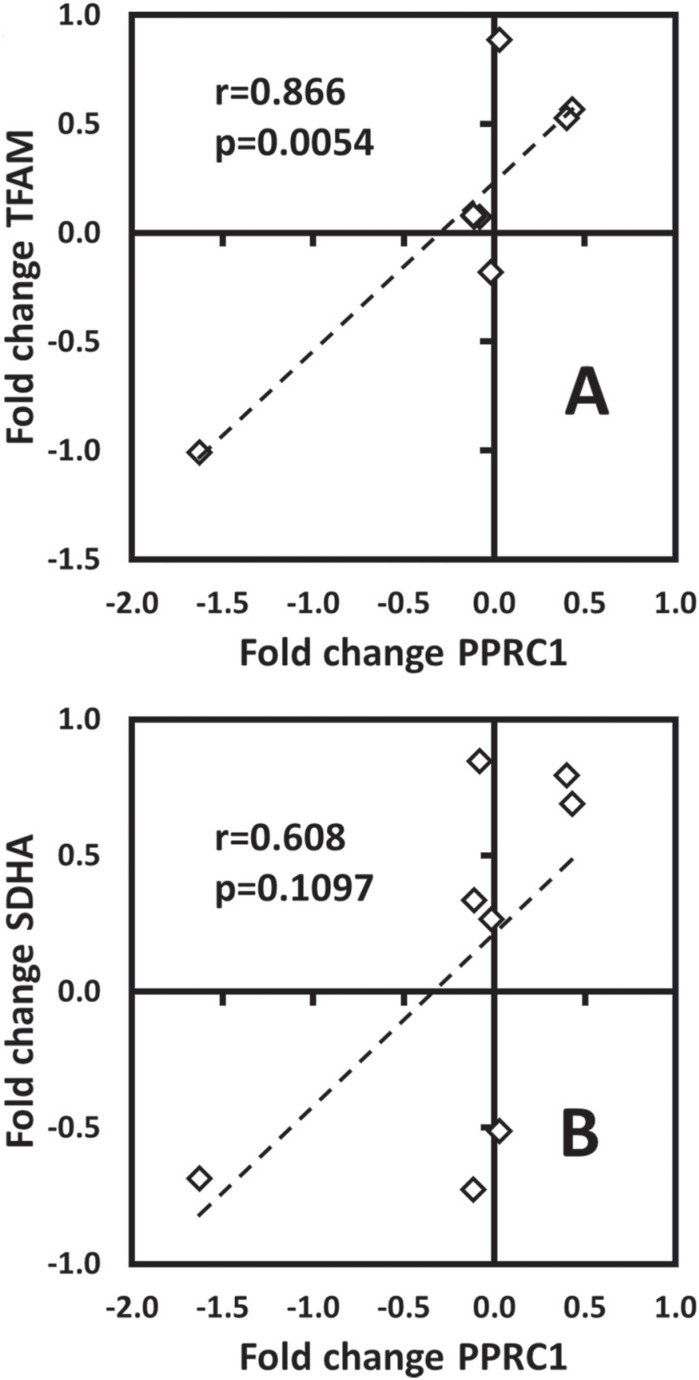
Pearson's correlation analysis of gene expression of PPRC1 vs. TFAM and PPRC1 vs. SDHA in 8 cell lines. The gene expressions from cell lines FDH98, FDH111, FDH113, XPHM16VI, XPHF16VI, XP17VI, XP4PA and AS860 were plotted in a scatter X&Y chart and Pearson's correlation (r_value_) was calculated. A) Pearson's correlation of gene expression between PPRC1 (*x*-axis) vs. TFAM (*y*-axis). B) Pearson's correlation of gene expression between PPRC1 (*x*-axis) vs. SDHA (*y*-axis). Control cell lines MRC-5 and FDH107 were excluded from the analysis owing to its biased reference values.

Thus, PPRC1 expression was measured by qRT-PCR with ten fibroblast cell lines (Figure 3) and we found that its expression closely followed that of TFAM and, to a lesser extent, SDHA (Figures 4A, B). Individual Pearson's correlation between PPRC1 vs. TFAM was 0.8662 (p=0.0054). Even though PPRC1 vs. SDHA correlation (r=0.6081) did not reach statistical significance (p=0.1097), it was much higher than individually calculated correlations between PGC-1α vs. TFAM or SDHA. Calculated multiple correlation coefficient between PPRC1, TFAM and SDHA gene expression was R=0.93 (very high), while for PGC-1α, TFAM and SDHA was R=0.49. Furthermore, adjusted multiple coefficient of determination between PPRC1, TFAM and SDHA gene expression was R^2^_adj_ =0.81 (high correlation) and PPRC1 expression varied much less between cell lines compared to PGC-1α (Figure S4). Lastly, PGC-1β expression was not detected with four different pairs of primers. These results indicate that PPRC1 contributes to a greater extent to the mitochondrial biogenesis program in fibroblasts.

### PPRC1 is stably expressed at protein levels in fibroblast and better correlates with PGC-1 target genes than PGC-1α

**Figure 5:**
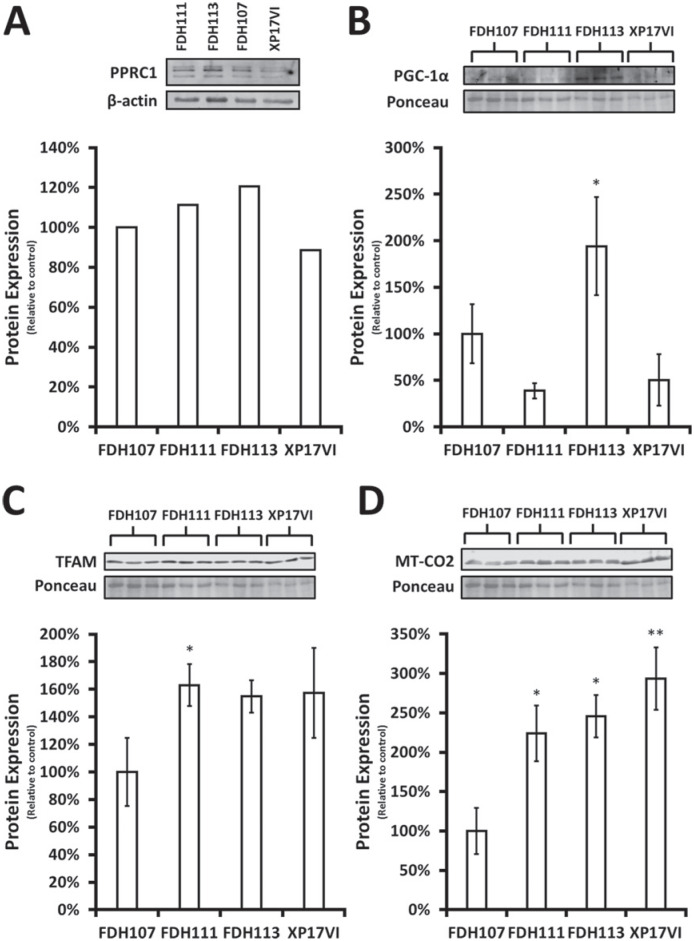
Analysis of PGC-1α, PPRC1, TFAM and MT-CO2 protein levels in 4 cell lines. Protein levels from four primary human fibroblast cell lines, FDH107, FDH111, FDH113 and XP17VI, were assessed by immunoblotting. A) Protein levels of PPRC1, B) PGC-1α (mean±SD, n=3), C) TFAM (mean±SD, n=3) and D) MT-CO2 (mean±SD, n=3). FDH107 was set as reference (100%). *p<0.05 and **p<0.01 compared to FDH107 cell line.

Because mRNA levels not always directly correlate with protein levels, we asked if the observed variation of PGC-1α mRNA levels would reflect in protein levels. We chose to analyze four human primary fibroblast cell lines: i) a control (FDH107), ii) a high expressing (FDH111), iii) a low expressing (FDH113) and iv) an XP-C cell line (XP17VI). Western blot showed that PGC-1α protein levels varied considerably between cell lines (Figure 5). However, mRNA levels did not correlate and, in some cases, inversely correlated with protein levels. On the other hand, the four cell lines stably expressed PPRC1 at protein levels compared to PGC-1α.

To check which PGC-1 family member functionally affects their target-genes, we performed immunoblot against TFAM as a read-out of PGC-1 activity. TFAM did not followed PGC-1α protein expression levels but showed a similar expression pattern to that of PPRC1 protein levels, with the exception of XP17VI cell line. To confirm that increased TFAM levels result in increased transcriptional activity of mitochondrial-encoded genes, we also investigated MT-CO2 expression. As expected, MT-CO2 levels correlated well with TFAM in all cell lines. Taken together, these results suggest that PPRC1 could act as a maintenance coactivator of the mitochondrial biogenesis program.

### PGC-1α knockdown affect TFAM expression and its target-gene MT-CO2

**Figure 6:**
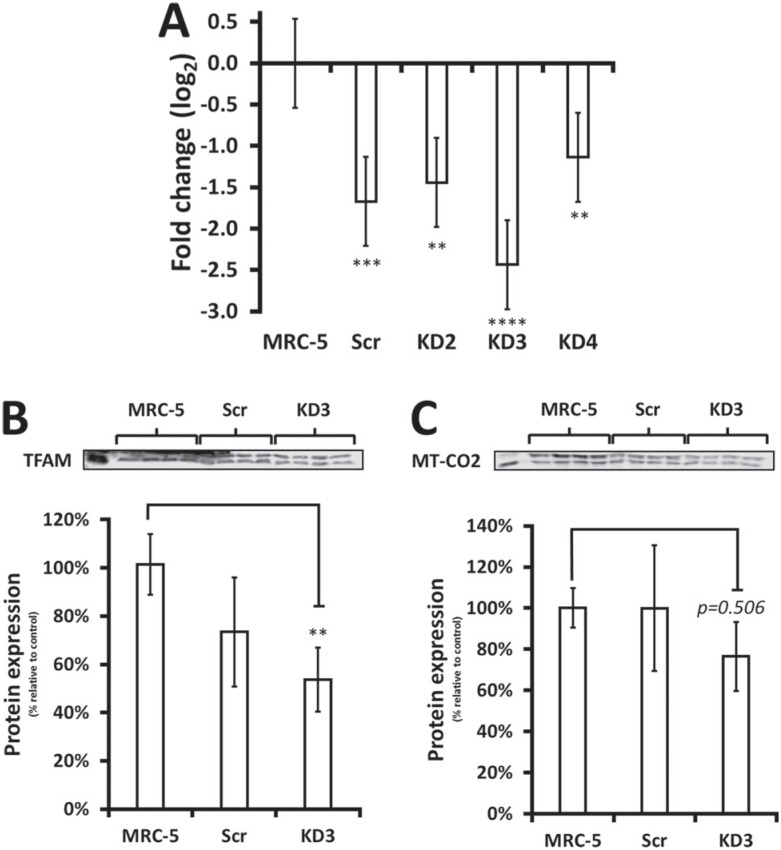
Analysis of PGC-1α knockdown impact in fibroblasts. A) PGC-1α mRNA expression of MRC-5 fibroblasts untransfected and knockdown cells were accessed by RT-qPCR (mean±SD, n=3). B) Protein levels of TFAM in untransfected MRC-5 cells (MRC-5), transfected scramble shRNA MRC-5 cells (Scr) and transfected PGC-1α knockdown MRC-5 cells (KD3). C) Protein levels of MT-CO2 in MRC-5, Scr and KD3 cells (mean±SD, n=4). MRC-5 was set as reference (100%). **p<0.01, ***p<0.001 and ****p<0.0001.

To test whether PGC-1α plays a minor role in fibroblasts, we stably transfected plasmids expressing shRNA targeting PGC-1α to induce knockdown in MRC-5 cells. Of all four shRNAs used, clone 3 (KD3) presented the best silencing (84.9±2.5% decrease) (Figure 6A). Unfortunately, the scramble control also showed a significant decreased in PGC-1α compared to mock-transfected MRC-5 cells.

Next, we also measured TFAM protein expression levels is this set up. Protein levels of TFAM were significantly decreased in PGC-1α KD cells compared to MRC-5 (Figure 6B). Furthermore, although MT-CO2 protein levels were not significantly reduced in PGC-α KD cells, there was a trend to lower expression (Figure 6C). This result suggests that PGC-1α knockdown in fibroblast interferes with expression of PGC-1 family target-gene, TFAM, and, consequently, of mtDNA genes, such as MT-CO2. Furthermore, activating PGC-1α expression via AICAR treatment, an AMPK activator, led to approximately 2-fold increase in PGC-1α expression in both MRC-5 and XP4PA fibroblasts (Figure S5). Taken together these results suggest that, despite lack of correlation with expression of mitochondrial genes, PGC-1α retains adaptive metabolic function in fibroblasts.

## DISCUSSION

In the present study we started investigating a highly reproducible phenomenon, of decreased PGC-1α expression in XP-C fibroblasts (Figures 1 and 2). Hypermethylation of the PGC-1α promoter has been proposed to modulate its expression in muscle samples from patients with type 2 diabetes (Barrès *et al.*, 2010). Nonetheless, an 8 day-treatment with the pan-demethylating agent 5-aza-dC did not changed expression of PGC-1α in XP-C^ΔTG/ΔTG^ cells nor its WT counterpart or XP-C^+/+^ isogenic cell line (Figure S1-C). In parallel to this experiment, XPC knockdown did not affected PGC-1α expression (Figure S1A), prompting us to speculate if the nature of *XPC* mutation could be involved in the reduced expression of PGC-1α.

A previous study demonstrated that a large deletion in α-globin cluster can create an antisense lncRNA that affects gene expression via DNA methylation (Tufarelli *et al.*, 2003). This mutation occurs juxtaposed to *LUC7L* gene that is expressed in the opposite direction of *HBA2*. Thus, *LUC7L* is transcribed invading *HBA2* gene, specially a CpG region. This transcript leads to CpG methylation-induced silencing of *HBA2* eliciting α-thalassemia phenotype. Furthermore, Yu *et al.* (2008) reported that overexpression of a natural antisense RNA, p15AS, controlled p15 expression via epigenetic histone modification silencing. This mechanism was DICER-independent and involved repressive heterochromatin formation within p15 promoter. Moreover, even after p15AS was no longer expressed, p15 promoter was still silenced. Therefore, we wondered if a similar phenomenon could be happening in our model, although we were aware that a lncRNA from *XPC* (located in Chr. 3) would need to act in *trans* in PGC-1α locus (located in Chr. 4).

Although all cell lines carrying the XPC^ΔTG^ allele displayed reduced PGC-1α levels (Figure 2), there was no correlation of that with expression of PGC-1 family target-genes, TFAM and SDHA (Figure 3), even though the PGC-1α locus was transcriptionally active, as it responded to AICAR stimulation (Figures S5). Instead, we found significant correlation only between PPRC1 expression and those genes (Figure 4). However, it should be noted that most mRNA and protein levels correlate poorly in most biological systems (de Sousa Abreu *et al.*, 2009; Vogel and Marcotte, 2012).

PPRC1 was first described by Andersson and Scarpulla (2001) as a transcriptional co-activator induced upon serum addition into culture medium of quiescent cells. This study showed that PPRC1 is ubiquitously expressed and, differently than PGC-1α, it is irresponsive to thermal stimulus. In fact, several lines of evidence support a basal role for PPRC1 in mitochondrial maintenance. The same group demonstrated that knocking down PPRC1 led to a severe decrease in mitochondrial mass and mtDNA copy number, decreased activity and expression of the electron transport chain complexes and impaired proliferative capacity in culture medium with galactose as the only source of carbon (Vercauteren *et al.*, 2009). These data suggest that PPRC1 acts as a housekeeping gene, contrary to PGC-1α and β that display a role in adaptative responses (Wu *et al.*, 1999)

Additional studies demonstrated that PPRC1 is a transcriptional co-activator for stress response programs, including redox stress and single strand DNA breaks. In all tested protocols, PPRC1 is simultaneously upregulated with transcription factor and proto-oncogene c-myc (Gleyzer and Scarpulla, 2013). While increased c-myc expression seemed to be regulated by PPRC1 in a feedback loop, PPRC1 is activated at a post-translational level through increased protein stability. The increased PPRC1 stability is regulated by two phosphorylation motifs by GSK3 kinase at its C-terminus, which also targets c-myc. AKT1 phosphorylates and inactivates GSK-3β, which, in turn, does not phosphorylate PPRC1, increasing its stability (Gleyzer and Scarpulla, 2016). Interestingly, PPRC1 is commonly found to be overexpressed in thyroid tumor, concomitant with increased activity of cytochrome oxidase and expression of some subunits of the respiratory chain (Savagner *et al.*, 2003; Raharijaona *et al.*, 2009).

Metabolic alteration in cancer have emerged as an important adaptative feature of neoplastic cells to bypass adverse situations (Pavlova and Thompson, 2016). In our laboratory we have been using XP-C cells as a model of genomic instability to investigate metabolic alterations arising from DNA repair dysfunction. In that regard, XP-C cells represent a clean genomic instability model, as mutations in other genes of the NER pathways may lead to far more complex phenotypes, with the combination of two or more syndromes (Cleaver *et al.*, 2009; DiGiovanna and Kraemer, 2012; Marteijn *et al.*, 2014). Because we previously described one phenomenon of mitochondrial complexes utilization shift in XP-C, and due to the marked decreased in PGC-1α expression in this cell line (Figure 1), we raised the possibility that these phenomena might be linked. The results reported here do not support the hypothesis that the decreased PGC-1α expression is mechanistically linked to the metabolic shift between mitochondrial complexes I and II observed in XP-C cells, as we did not find a direct correlation between PGC-1α levels and SDHA expression in XP-C cells (Figure S2). However, PGC-1α knockdown, via shRNA, decreased TFAM and MT-CO2 protein levels (Figure 6), such that we cannot exclude PGC-1α involvement in control of mtDNA expression.

In this current study, we observed that PPRC1 protein and mRNA levels better correlated with some target genes of the PGC-1 family than PGC-1α, at least in the cell lines used. PPRC1 correlated with TFAM, which, in turn, correlated well with MT-CO2. XP17VI was the only of the four human primary cell lines tested that did not show correlation between PPRC1 and TFAM. Our group has been investigating metabolic alterations in XP-C cell lines. PPRC1 was significantly up regulated in the transformed XP-C fibroblast cell line, XP4PA, compared to MRC-5, but not in primary fibroblasts, suggesting that another signaling axis is likely to be acting in this context. Ongoing investigation from our group indicates that the DNA damage response factor, p53, might be this factor (unpublished data).

Our data also do not support the hypothesis that XPC protein levels or a specific mutation in the *XPC* gene are responsible for PGC-1α downregulation in XP-C cells, although we cannot completely exclude that due to the high variation in its expression observed in the cell lines used. On the other hand, our results support previous findings that PPRC1 may have a significant role in maintaining basal mitochondrial function in fibroblast cell lines in culture and should be considered as a more relevant transcriptional co-activator when using this model.

## Supplementary material

The following online material is available for this article:

Figure S1 - Analysis of PGC-1α silencing mechanism.

Figure S2 - Impact of PGC-1α expression on mitochondrial function.

Figure S3 - Gene expression data of PGC-1 family of transcriptional co-activators genes in GTEx Portal.

Figure S4 - Analysis of radial symmetry distribution of PGC-1α, PPRC1, TFAM and SDHA gene expression in human primary fibroblasts.

Figure S5 - Analysis of PGC-1α accessibility by AMPK activation.

Table S1 - Genes list used to investigate gene expression by RT-qPCR.

## References

[B1] Andersson U, Scarpulla RC (2001). Pgc-1-related coactivator, a novel, serum-inducible coactivator of nuclear respiratory factor 1-dependent transcription in mammalian cells. Mol Cell Biol.

[B2] Abreu RS, Penalva LO, Marcotte EM, Vogel C (2009). Global signatures of protein and mRNA expression levels. Mol Biosyst.

[B3] Barrès R, Osler ME, Yan J, Rune A, Fritz T, Caidahl K, Krook A, Zierath JR (2010). Non-CpG methylation of the PGC-1alpha promoter through DNMT3B controls mitochondrial density. Cell Metab.

[B4] Chen X, Velmurugu Y, Zheng G, Park B, Shim Y, Kim Y, Liu L, Van Houten B, He C, Anjum A (2015). Kinetic gating mechanism of DNA damage recognition by Rad4/XPC. Nat Commun.

[B5] Cleaver JE, Lam ET, Revet I (2009). Disorders of nucleotide excision repair: the genetic and molecular basis of heterogeneity. Nat Rev Genet.

[B6] Cortázar D, Kunz C, Selfridge J, Lettieri T, Saito Y, MacDougall E, Wirz A, Schuermann D, Jacobs AL, Siegrist F (2011). Embryonic lethal phenotype reveals a function of TDG in maintaining epigenetic stability. Nature.

[B7] D’Errico M, Parlanti E, Teson M, Jesus BM, Degan P, Calcagnile A, Jaruga P, Bjørås M, Crescenzi M, Pedrini AM (2006). New functions of XPC in the protection of human skin cells from oxidative damage. EMBO J.

[B8] DiGiovanna JJ, Kraemer KH (2012). Shining a light on xeroderma pigmentosum. J Invest Dermatol.

[B9] Dupuy A, Valton J, Leduc S, Armier J, Galetto R, Gouble A, Lebuhote C, Stary A, Pâques F, Duchateau P (2013). Targeted gene therapy of xeroderma pigmentosum cells using meganuclease and TALEN. PLoS One.

[B10] Fong YW, Inouye C, Yamaguchi T, Cattoglio C, Grubisic I, Tjian RA (2011). DNA repair complex functions as an Oct4/Sox2 coactivator in embryonic stem cells. Cell.

[B11] Gleyzer N, Scarpulla RC (2013). Activation of a PGC-1-related coactivator (PRC)-dependent inflammatory stress program linked to apoptosis and premature senescence. J Biol Chem.

[B12] Gleyzer N, Scarpulla RC (2016). Concerted action of PGC-1-related coactivator (PRC) and c-MYC in the stress response to mitochondrial dysfunction. J Biol Chem.

[B13] Hansford RG, Castro F (1982). Age-linked changes in the activity of enzymes of the tricarboxylate cycle and lipid oxidation, and of carnitine content, in muscles of the rat. Mech Ageing Dev.

[B14] Ho JJ, Cattoglio C, McSwiggen DT, Tjian R, Fong YW (2017). Regulation of DNA demethylation by the XPC DNA repair complex in somatic and pluripotent stem cells. Genes Dev.

[B15] Ichida M, Nemoto S, Finkel TJ (2002). Identification of a specific molecular repressor of the peroxisome proliferator-activated receptor gamma coactivator-1 alpha (PGC-1alpha). J Biol Chem.

[B16] Leite RA, Marchetto MC, Muotri AR, Vasconcelos DM, Oliveira ZN, Machado MC, Menck CF (2009). Identification of XP complementation groups by recombinant adenovirus carrying DNA repair genes. J Invest Dermatol.

[B17] Le May N, Mota-Fernandes D, Vélez-Cruz R, Iltis I, Biard D, Egly JM (2010). NER factors are recruited to active promoters and facilitate chromatin modification for transcription in the absence of exogenous genotoxic attack. Mol Cell.

[B18] Liang G, Gonzales FA, Jones PA, Orntoft TF, Thykjaer T (2002). Analysis of gene induction in human fibroblasts and bladder cancer cells exposed to the methylation inhibitor 5-aza-2’-deoxycytidine. Cancer Res.

[B19] Marteijn JA, Lans H, Vermeulen W, Hoeijmakers JH (2014). Understanding nucleotide excision repair and its roles in cancer and ageing. Nat Rev Mol Cell Biol.

[B20] Min JH, Pavletich NP (2007). Recognition of DNA damage by the Rad4 nucleotide excision repair protein. Nature.

[B21] Mori MP, Costa RA, Soltys DT, Freire TS, Rossato FA, Amigo I, Kowaltowski AJ, Vercesi AE, Souza-Pinto NC (2017). Lack of XPC leads to a shift between respiratory complexes I and II but sensitizes cells to mitochondrial stress. Sci Rep.

[B22] Neter J, Kutner MH, Nachtsheim C, Wasserman W (1996). Applied Linear Statistical Models.

[B23] Pavlova NN, Thompson CB (2016). The emerging hallmarks of cancer metabolism. Cell Metab.

[B24] Pfaffl MW (2001). A new mathematical model for relative quantification in real-time RT-PCR. Nucleic Acids Res.

[B25] Raharijaona M, Le Pennec S, Poirier J, Mirebeau-Prunier D, Rouxel C, Jacques C, Fontaine JF, Malthiery Y, Houlgatte R, Savagner F (2009). PGC-1-related coactivator modulates mitochondrial-nuclear crosstalk through endogenous nitric oxide in a cellular model of oncocytic thyroid tumours. PLoS One.

[B26] Rezvani HR, Kim AL, Rossignol R, Ali N, Daly M, Mahfouf W, Bellance N, Taïeb A, de Verneuil H, Mazurier F (2011). XPC silencing in normal human keratinocytes triggers metabolic alterations that drive the formation of squamous cell carcinomas. J Clin Invest.

[B27] Rivera-Begeman A, McDaniel LD, Schultz RA, Friedberg EC (2007). A novel XPC pathogenic variant detected in archival material from a patient diagnosed with Xeroderma Pigmentosum: a case report and review of the genetic variants reported in XPC. DNA Repair (Amst).

[B28] Savagner F, Mirebeau D, Jacques C, Guyetant S, Morgan C, Franc B, Reynier P, Malthièry Y (2003). PGC-1-related coactivator and targets are upregulated in thyroid oncocytoma. Biochem Biophys Res Commun.

[B29] Shimizu Y, Iwai S, Hanaoka F, Sugasawa K (2003). Xeroderma pigmentosum group C protein interacts physically and functionally with thymine DNA glycosylase. EMBO J.

[B30] Shimizu Y, Uchimura Y, Dohmae N, Saitoh H, Hanaoka F, Sugasawa K (2010). Stimulation of DNA glycosylase activities by XPC protein complex: Roles of protein-protein interactions. J Nucleic Acids.

[B31] Soufir N, Ged C, Bourillon A, Austerlitz F, Chemin C, Stary A, Armier J, Pham D, Khadir K, Roume J (2010). A prevalent mutation with founder effect in xeroderma pigmentosum group C from north Africa. J Invest Dermatol.

[B32] Sugasawa K, Ng JM, Masutani C, Iwai S, Van Der Spek PJ, Eker AP, Hanaoka F, Bootsma D, Hoeijmakers JH (1998). Xeroderma pigmentosum group C protein complex is the initiator of global genome nucleotide excision repair. Mol Cell.

[B33] Tufarelli C, Stanley JAS, Garrick D, Sharpe JA, Ayyub H, Wood WG, Higgs DR (2003). Transcription of antisense RNA leading to gene silencing and methylation as a novel cause of human genetic disease. Nat Genet.

[B34] Uribe-Bojanini E, Hernandez-Quiceno S, Cock-Rada AM (2017). Xeroderma pigmentosum with severe neurological manifestations/De Sanctis-Cacchione syndrome and a novel XPC mutation. Case Rep Med.

[B35] Vercauteren K, Gleyzer N, Scarpulla R (2009). Short hairpin RNA-mediated silencing of PRC (PGC-1-related coactivator) results in a severe respiratory chain deficiency associated with the proliferation of aberrant mitochondria. J Biol Chem.

[B36] Vogel C, Marcotte EM (2012). Insights into the regulation of protein abundance from proteomic and transcriptomic analyses. Nat Rev Genet.

[B37] Volker M, Mone MJ, Karmakar P, Van Hoffen A, Schul W, Vermeulen W, Hoeijmakers JH, van Driel R, van Zeeland AA, Mullenders LH (2001). Sequential assembly of the nucleotide excision repair factors *In vivo*. Mol Cell.

[B38] Yu W, Gius D, Onyango P, Muldoon-Jacobs K, Karp J, Feinberg AP, Cui H (2008). Epigenetic silencing of tumour suppressor gene p15by its antisense RNA. Nature.

[B39] Wu Z, Puigserver P, Andersson U, Zhang C, Adelmant G, Mootha V, Troy A, Cinti S, Lowell B, Scarpulla RC (1999). Mechanisms controlling mitochondrial biogenesis and respiration through the thermogenic coactivator PGC-1. Cell.

