# Supplementary material for: PPRC1, but not PGC-1α, levels directly correlate with expression of mitochondrial proteins in human dermal fibroblasts
Source: Genet Mol Biol. 2020 Jul 3;43(1 Suppl 1):e20190083. doi: 10.1590/1678-4685-GMB-2019-0083 (PMC7341727; doi:10.1590/1678-4685-GMB-2019-0083)
Supplement: Supplementary file 1 [file 1415-4757-GMB-43-1-s1-e20190083-s6.pdf]

Supplementary material to "PPRC1, but not PGC-1*α*, levels directly correlate with expression of mitochondrial proteins in human dermal fibroblasts"

Table S1. List of genes used to investigate gene expression by RT-qPCR.

ACTB, TBP, TUBB and HPRT were used as reference housekeeping genes.

Gene	Gene Name	Sequence		mRNA RefSeq
NRF1	nuclear respiratory factor 1	Fw	CTC TGA GAA CTT CAT GGA GGA ACA	NM_005011.3
		Rev	CGT GTC CTC GGG AGA AGA AG	
NFE2L1	nuclear factor, erythroid 2-like 1	Fw	TAC TCC CAG GTT GCC CAC A	NM_006164.4
		Rev	CAT CTA CAA ACG GGA ATG TCT GC	
PPARα (*PPARA*)	peroxisome proliferator-activated receptor alpha	Fw	GTC TCC CAG RGG AGC ATT GA	NM_005036.4
		Rev	ACC AGC TTG AGT CGA ATC GT	
AMPK (*PRKAA1*)	protein kinase, AMP-activated alpha 1 catalytic subunit	Fw	CGG AGC CTT GAT GTG GTA GG	NM_006251.5
		Rev	TCA TCC AGC CTT CCA TTC TTA CA	
SIRT1	sirtuin 1	Fw	TGG GTA CCG AGA TAA CCT TCT	NM_012238.4
		Rev	TGT TCG AGG ATC TGT GCC AA	
SIRT3	sirtuin 3	Fw	CAC AGT CTG CCA AAG ACC CT	NM_012239.5
		Rev	CAA TGT CGG GCT TCA CAA CG	
SDHA	succinate dehydrogenase complex, subunit A, flavoprotein (Fp)	Fw	CCT TTC TGA GGC AGG GTT TA	NM_004168.2
		Rev	AGA GCA GCA TTG ATT CCT CC	
PGC-1α (*PPARGC1A*)	peroxisome proliferator-activated receptor gamma, coativator 1 alpha	Fw	AGA CAC CGC ACG CAC CGA AAT	NM_013261.3
		Rev	AGC TGT CAT ACC TGG GCC GAC G	
PPRC1	peroxisome proliferator-activated receptor gamma, coativator related 1	Fw	GAG CAG GTT ATC TCT GGA GGA	NM_015062.4
		Rev	AGC AGC TCC GAA TCA GGA ATG	
TFAM	transcription factor A, mitochondrial	Fw	AAC CAA AAA GAC CTC GTT CAG C	NM_003201.2
		Rev	TTC AGC TTT TCC TGC GGT GA	
KRT8	keratin 8, type II	Fw	GAA TGA ATG GGG TGA GCT GGA	NM_001256282.1
		Rev	AGG TGG ACA CCT TGT AGG ACT	
**Gene**	**Gene Name**		**Sequence**	**mRNA RefSeq**

XPC	xeroderma pigmentosum, complementation group C	Fw	CAT CGT GGG AGC CAT CGT AAG	NM_004628.4
		Rev	CTC ACC ATC CGC TGC ACA TTT T	
ACTB	actin, beta	Fw	CTC TTC CAG CCT TCC TTC CT	NM_001101.3
		Rev	AGC ACT GTG TTG GCG TAC AG	
TUBB	tubulin, beta class I	Fw	TGG ACT CTG TTC GCT CAG GT	NM_001293212.1
		Rev	TGC CTC CTT CCG TAC CAC AT	
TBP	TATA box binding protein	Fw	CCA CTC ACA GAC TCT CAC AAC	NM_003194.4
		Rev	CTG CGG TAC AAT CCC AGA ACT	
HPRT	hypoxanthine phosphorybosyltransferase	Fw	GAA AAG GAC CCC ACG AAG TGT	NM_000194.2
		Rev	AGT CAA GGG CAT ATC CTA CAA CA	
