# Supplementary material for: PPRC1, but not PGC-1α, levels directly correlate with expression of mitochondrial proteins in human dermal fibroblasts
Source: Genet Mol Biol. 2020 Jul 3;43(1 Suppl 1):e20190083. doi: 10.1590/1678-4685-GMB-2019-0083 (PMC7341727; doi:10.1590/1678-4685-GMB-2019-0083)
Supplement: Supplementary file 2 [file 1415-4757-GMB-43-1-s1-e20190083-s1.pdf]

Supplementary material to "PPRC1, but not PGC-1*α*, levels directly correlate with expression of mitochondrial proteins in human dermal fibroblasts"

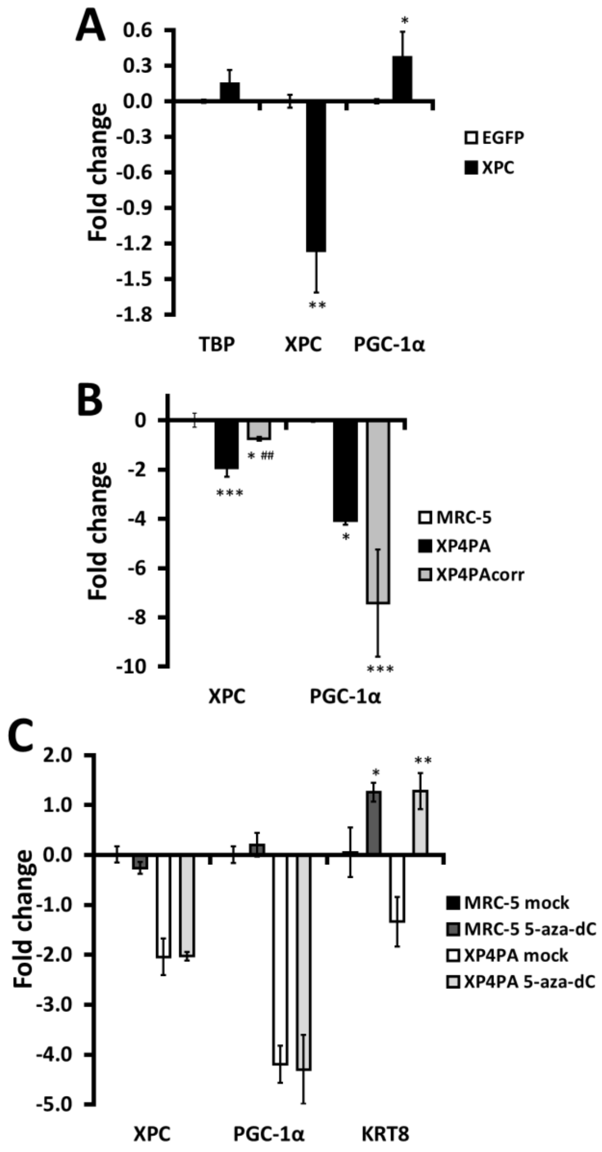

Figure S1. Analysis of PGC-1α silencing mechanism.

A) TBP, PGC-1α and XPC mRNA expression in MRC-5 fibroblasts transiently transfected with esiRNA targeting EGFP (control) and XPC (mean±SD, n=3). **B)** XPC and PGC-1α mRNA expression in MRC-5, XP4PA and XP4PA corrected with WT XPC *in locus* (XP4PAcorr) (mean±SD, n=3). **C)** XPC, PGC-1α and KRT8 mRNA expression in MRC-5 and XP4PA treated with 5-aza-dC Substitute 10 mM for 1 uM. for 8 days and mock-treated cells (control) (mean±SD, n=3). *p<0.05, **p<0.01 and ***p<0.001 compared to the control. ##p<0.01 compared to XP4PA.
