# Supplementary material for: PPRC1, but not PGC-1α, levels directly correlate with expression of mitochondrial proteins in human dermal fibroblasts
Source: Genet Mol Biol. 2020 Jul 3;43(1 Suppl 1):e20190083. doi: 10.1590/1678-4685-GMB-2019-0083 (PMC7341727; doi:10.1590/1678-4685-GMB-2019-0083)
Supplement: Supplementary file 3 [file 1415-4757-GMB-43-1-s1-e20190083-s2.pdf]

Supplementary material to PPRC1, but not PGC-1*α*, levels directly correlate with expression of mitochondrial proteins in human dermal fibroblasts

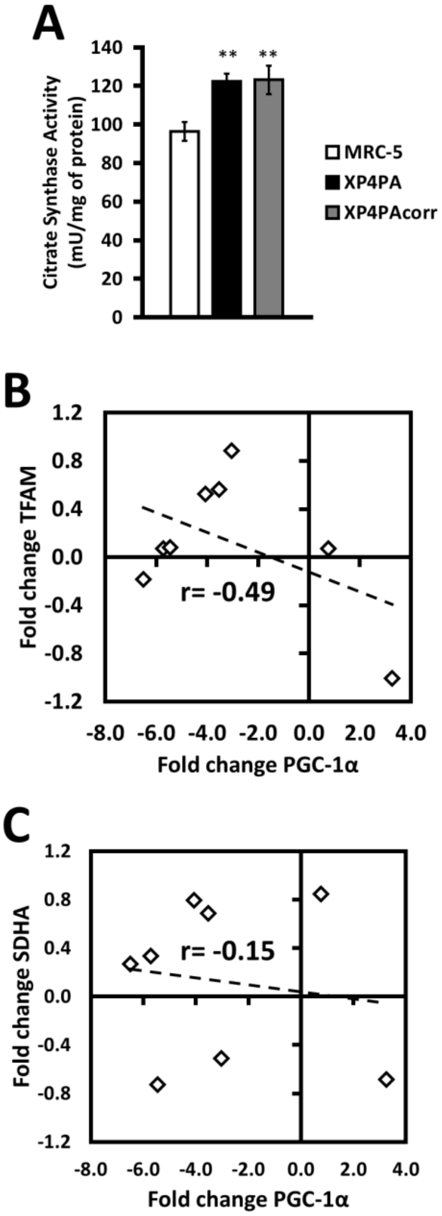

Figure S2. Impact of PGC-1α expression on mitochondrial function.

A) Citrate synthase activity in MRC-5, XP4PA and XP4PAcorr cells. **B)** Pearson's correlation of gene expression between PGC-1α (*x*-axis) vs. TFAM (*y*-axis). **C)** Pearson's correlation of gene expression between PGC-1α (*x*-axis) vs. SDHA (*y*-axis). Each cell line gene expression was plotted in a scatter X&Y chart and Pearson's correlation (r_{value}) was calculated. Control cell lines MRC-5 and FDH107 were excluded from the analysis owing to its biased reference values p<0.01.
