# Supplementary material for: PPRC1, but not PGC-1α, levels directly correlate with expression of mitochondrial proteins in human dermal fibroblasts
Source: Genet Mol Biol. 2020 Jul 3;43(1 Suppl 1):e20190083. doi: 10.1590/1678-4685-GMB-2019-0083 (PMC7341727; doi:10.1590/1678-4685-GMB-2019-0083)
Supplement: Supplementary file 4 [file 1415-4757-GMB-43-1-s1-e20190083-s3.pdf]

Supplementary material to "PPRC1, but not PGC-1α, levels directly correlate with expression of mitochondrial proteins in human dermal fibroblasts"

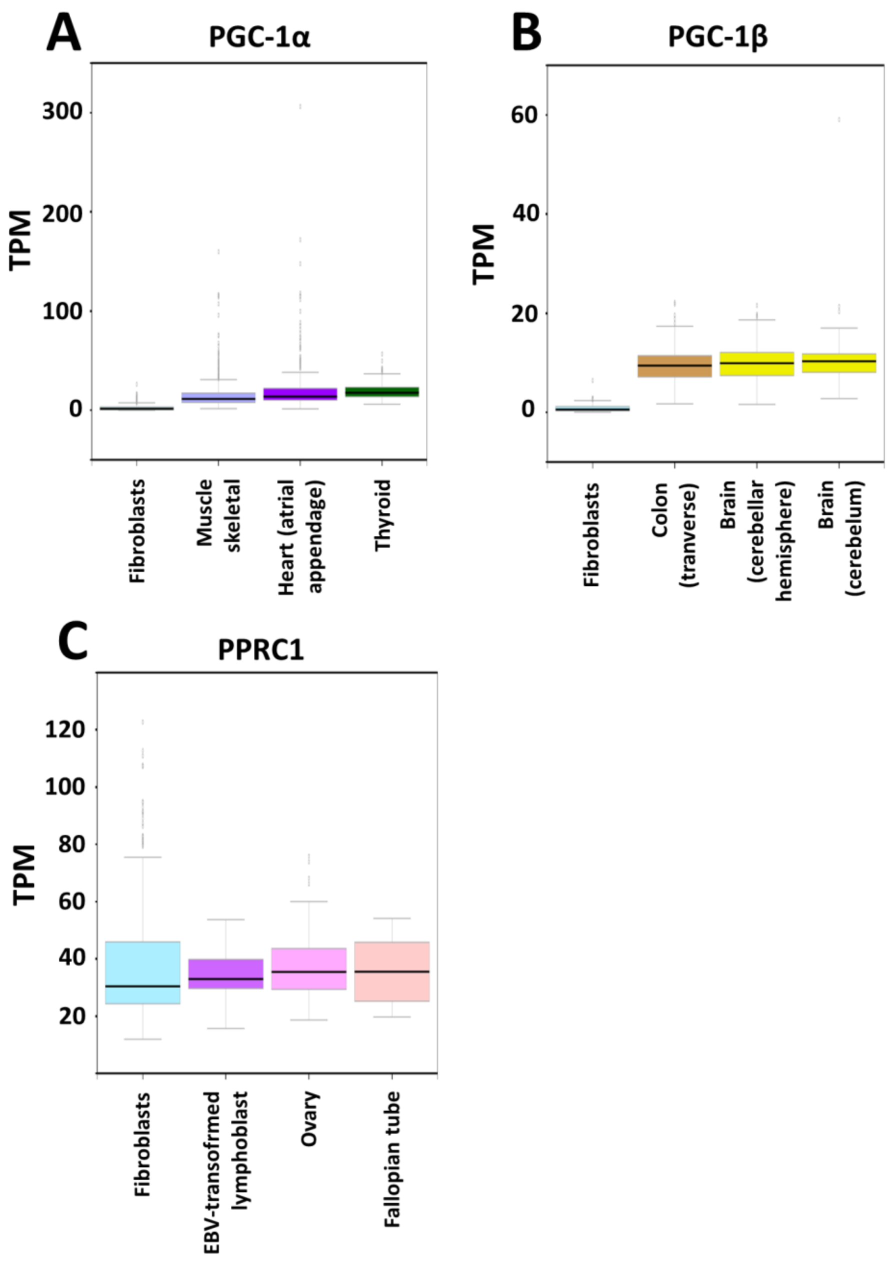

Figure S3. Gene expression data of PGC-1 family of transcriptional co-activators genes in GTEx Portal.

The search was performed in the website https://gtexportal.org for the following genes (A) PGC-1α (*PPARGC1A*), (B) PGC-1β (*PPARGC1A*) e (C) PPRC1. Exported graphs contain top 3 expressing cell types, tissues and organs in comparison to fibroblasts for each gene (of a total of 53 cell types, tissues and organ collection).
