# Supplementary material for: PPRC1, but not PGC-1α, levels directly correlate with expression of mitochondrial proteins in human dermal fibroblasts
Source: Genet Mol Biol. 2020 Jul 3;43(1 Suppl 1):e20190083. doi: 10.1590/1678-4685-GMB-2019-0083 (PMC7341727; doi:10.1590/1678-4685-GMB-2019-0083)
Supplement: Supplementary file 5 [file 1415-4757-GMB-43-1-s1-e20190083-s4.pdf]

Supplementary material to "PPRC1, but not PGC-1*α*, levels directly correlate with expression of mitochondrial proteins in human dermal fibroblasts"

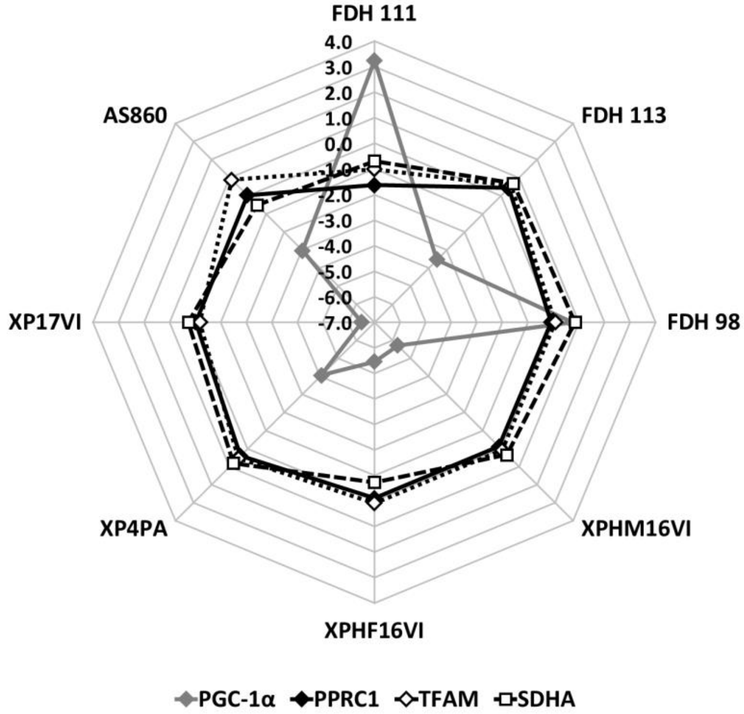

Figure S4. Analysis of radial symmetry distribution of PGC-1α, PPRC1, TFAM and SDHA gene expression in human primary fibroblasts**.**

Note that PPRC1, TFAM and SDHA gene expression level in each cell type closely follows each other and it is somewhat symmetrically distributed. PGC-1 gene expression does not follow any other gene and it is asymmetrically distributed.
