# Supplementary material for: PPRC1, but not PGC-1α, levels directly correlate with expression of mitochondrial proteins in human dermal fibroblasts
Source: Genet Mol Biol. 2020 Jul 3;43(1 Suppl 1):e20190083. doi: 10.1590/1678-4685-GMB-2019-0083 (PMC7341727; doi:10.1590/1678-4685-GMB-2019-0083)
Supplement: Supplementary file 6 [file 1415-4757-GMB-43-1-s1-e20190083-s5.pdf]

Supplementary material to "PPRC1, but not PGC-1*α*, levels directly correlate with expression of mitochondrial proteins in human dermal fibroblasts"

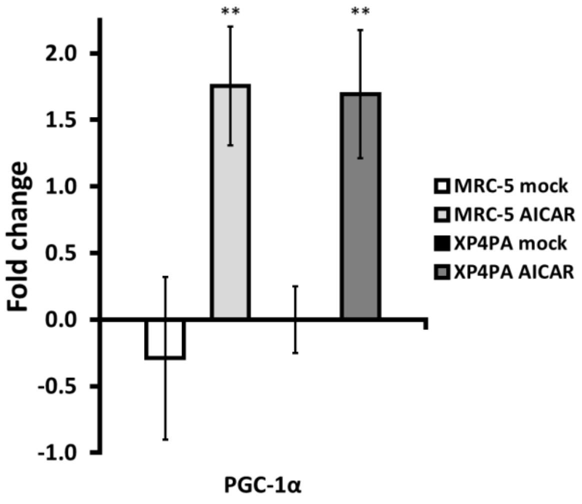

Figure S5. Analysis of PGC-1α accessibility by AMPK activation.

MRC-5 and XP4PA cell lines were treated with AICAR 1 mM and PGC-1α expression was measured as described. In both cell lines AICAR treatment was able to induce PGC-1α expression. $^{**}p<0.01$
